# Electrocardiographic changes in young patients with spontaneous pneumothorax: A retrospective study

**DOI:** 10.1097/MD.0000000000026793

**Published:** 2021-07-30

**Authors:** Baruch Klin, Itai Gueta, Haim Bibi, Shaul Baram, Ibrahim Abu-Kishk

**Affiliations:** a Pediatric Division, Shamir Medical Center (Assaf Harofeh), Zerifin, affiliated to the Sackler Faculty of Medicine, Tel-Aviv University, Tel-Aviv, Israel; b The Institute of Clinical Pharmacology, Sheba Medical Center, Tel Hashomer, Affiliated to the Sackler School of Medicine, Tel Aviv University, Tel Aviv, Israel.

**Keywords:** chest pain, dyspnea, electrocardiography, pediatric, primary spontaneous pneumothorax

## Abstract

Primary spontaneous pneumothorax (PSP) commonly occurs in adolescents. PSP symptoms can mimic cardiac event. We aimed to examine electrocardiography (ECG) changes that accompanied PSP in relation to side and size of pneumothorax.

A retrospectively reviewed 57 adolescents presented with PSP and underwent a cardiac evaluation.

Overall, 49 patients (86%) were male, median age of 16 years. Of these, 1 patient had a known mitral valve prolapse. In 56 patients the initial episode of PSP was unilateral (16 left sided and 40 right sided), and 1 was bilateral. The main initial symptom was chest pain or dyspnea and chest pain 66.6% and 33.3% respectively. Small pneumothorax was right and left sided in 1and 8 patients respectively, medium right (n = 8) medium left (n = 22), large right (n = 7) and large left (n = 10). One additional patient had medium bilateral pneumothorax. ECG findings were abnormal in 12 patients (21%) and included ST elevation in 5 patients, inverted T wave in 2 patients, incomplete right bundle branch block in 2 patients, poor R wave progression, left axis deviation and low QRS voltage in 1 patient each. Only 2 patients had abnormal echocardiography findings, MPV (n = 1) and minimal mitral and tricuspid regurgitation (n = 1). Serum troponin-T levels were normal in all patients.

ECG changes were found in 21% among pediatric patients with PSP. No correlation was observed between ECG changes and side/size of pneumothorax. It is important to rule out pneumothorax among children presented with chest pain, dyspnea and ECG changes.

## 1 Introduction

Primary spontaneous pneumothorax (PSP) most commonly occurs in young patients with no apparent underlying lung disease.^[1]^ Chest pain and sometimes breathlessness are the usual predominant presenting features of PSP.^[2]^ These symptoms can mimic a cardiac event; therefore, some patients who visit the emergency room with PSP undergo a cardiac examination. Electrocardiographic (ECG) is one of the most important diagnostic tools used in the differential diagnosis of these conditions. In the majority of cases, ECG abnormalities suggest cardiac problems. However, ECG changes with both right and left pneumothoraxes were previously reported. ECG changes that may occur with left-side pneumothorax include rightward shift in the mean frontal QRS axis, reduced precordial R wave amplitude, decreased QRS amplitude, precordial T wave inversion and phasic voltage alteration associated with respiration.^[3–8]^ Other reports of ECG changes accompanied left sided pneumothorax were suggestive of anterior myocardial infarction.^[4–10]^ Although, an abnormal axis is more common in patients with left-sided pneumothorax than in those with right-sided pneumothorax, while changes in QRS morphology such as right bundle branch block and inversion of T waves appear more often in patients with right-sided pneumothorax. The latter finding was only observed in patients with massive pneumothorax.^[11]^

In the present study, we reviewed pediatric patients who presented at our medical center with the final diagnosis of PSP and underwent a cardiac evaluation.

We aimed to examine changes in ECG, echocardiography and serum Troponin-T levels that accompanied PSP and their relation to the side and size of pneumothorax.

## 2 Methods

The study was approved by the local Ethics Committee of the Assaf Harofeh Medical Center. A retrospective review of the medical records of children with PSP that were admitted to Assaf Harofeh Medical Center from January 2000 to March 2020 was performed. In order to obtain a relatively uniform group, pediatric patients with underlying lung diseases, malignancy, infection, connective tissue disease, or congenital lung and heart diseases were excluded. Patients who did not undergo a cardiac evaluation were also excluded. Children with mild intermittent asthma with no limitations on activities were not excluded.

Demographic characteristics (age, gender, weight and height, history of smoking, and comorbidities), symptoms and signs on presentation, radiologic findings, initial and subsequent management, length of hospitalization and outcome were obtained.

The involved patients underwent a cardiac evaluation including ECG, echocardiography and serum Troponin T levels. In our medical center, serum Troponin-T levels <0.03 ng/mL are considered normal and levels >0.1 ng/mL indicate myocardial injury.

The quantification of pneumothorax size (%) was calculated by measuring interpleural distance on an erect postero-anterior chest radiograph, this method was previously reported.^[12]^ The management of PSP followed the institution protocol: small (<20%) pneumothoraxes are treated with high output oxygen therapy; medium to large pneumothoraxes (>20%) are treated with chest tube drainage. Pneumothorax dimension of 20% to 50% and >50% were considered medium and large respectively. Hypoxemia was evaluated by using a standard pulse oximeter, patient with a saturation value <94% was considered hypoxic and treated with oxygen supplement through a mask.^[13]^

The following ECG parameters were evaluated: QRS amplitudes in V1-V6, height and depth of R and S waves in V1-V2 and V5-V6, and position of the first lead counting from V1 to V6 with R wave higher than the depth of the S wave. Normal QRS amplitude was defined as the mean amplitude in V1-V6. Four ECG parameters were evaluated: heart rate, morphology of P waves, QRS complexes and T waves, axis deviation, QRS amplitude in precordial leads. Axis deviation in the frontal plane was determined using triaxial system of bipolar extremity lead.^[14]^

The extracted data about demographic and clinical signs, side and size of pneumothorax, ECG and echocardiography findings were compared.

### 2.1 Statistical analysis

Statistical analyzes were performed using SPSS software (version 25). Descriptive statistics were used to describe the study population results. Comparisons between groups (with /without ECG changes) were conducted using the Mann–Whitney *U* test for continuous, and Chi-Squared or Fisher exact test for categorical variables. Data are presented as mean ± standard deviation or median with interquartile range as appropriate for continuous variables and proportions for categorical variables. Missing data were excluded from the analyzes. All analyzes were two-tailed and *P* value of less than .05 was considered significant.

## 3 Results

Between 2000 and 2020, 109 children presented to the Assaf Harofeh Pediatric Surgery Department with PSP. Out of 109 patients, only 57 (52.3%) underwent a cardiac evaluation. The characteristics of the participants are illustrated in Table 1.

**Table 1:** Patients characteristics.

	Patients number (%)
Patients (%)	57 (100)
Median age in yr (range percentiles)	16 (15–17)
Mal gender (%)	49 (86)
Female gender (%)	8 (14)
Normal Medical background (%)	52 (91.29)
Smokers (%)	7 (12.2)
Left pneumothorax (%)	40 (70.1)
Right pneumothorax (%)	16 (28.1)
Bilateral pneumothorax (%)	1 (1.8)
Pneumothorax size (%):	
<20%	11 (19.3)^∗^
20%–50%	30 (52.6)^α^
>50%	16 (28.1)^β^
Abnormal ECG	12 (21%)
Abnormal ECHO^€^	2 (4.5)
Abnormal troponin	0 (0)
Presentation symptoms:	
Chest pain (%)	38 (66.6)
Chest pain & dyspnea (%)	19 (33.3%)
Median hospitalization days (range)	5 (4–8)
Median follow up in months (IQR)	3.5 (1–28)

Overall, 49 patients (86%) were male, and the median age at presentation was 16 (15–17) years. Of these children, 7 boys (12.2%) had a history of smoking, 6 children (10.5%) had underlying asthma, and 1 patient had mitral valve prolapse. No features of Marfan's syndrome were recorded. In 56 patients the initial episode of PSP was unilateral (16 left sided and 40 right sided), being bilateral in only one. The main initial symptom in 66.6% of the cases was chest pain alone followed by both dyspnea and chest pain in 33.3%.

**Figure 1:**
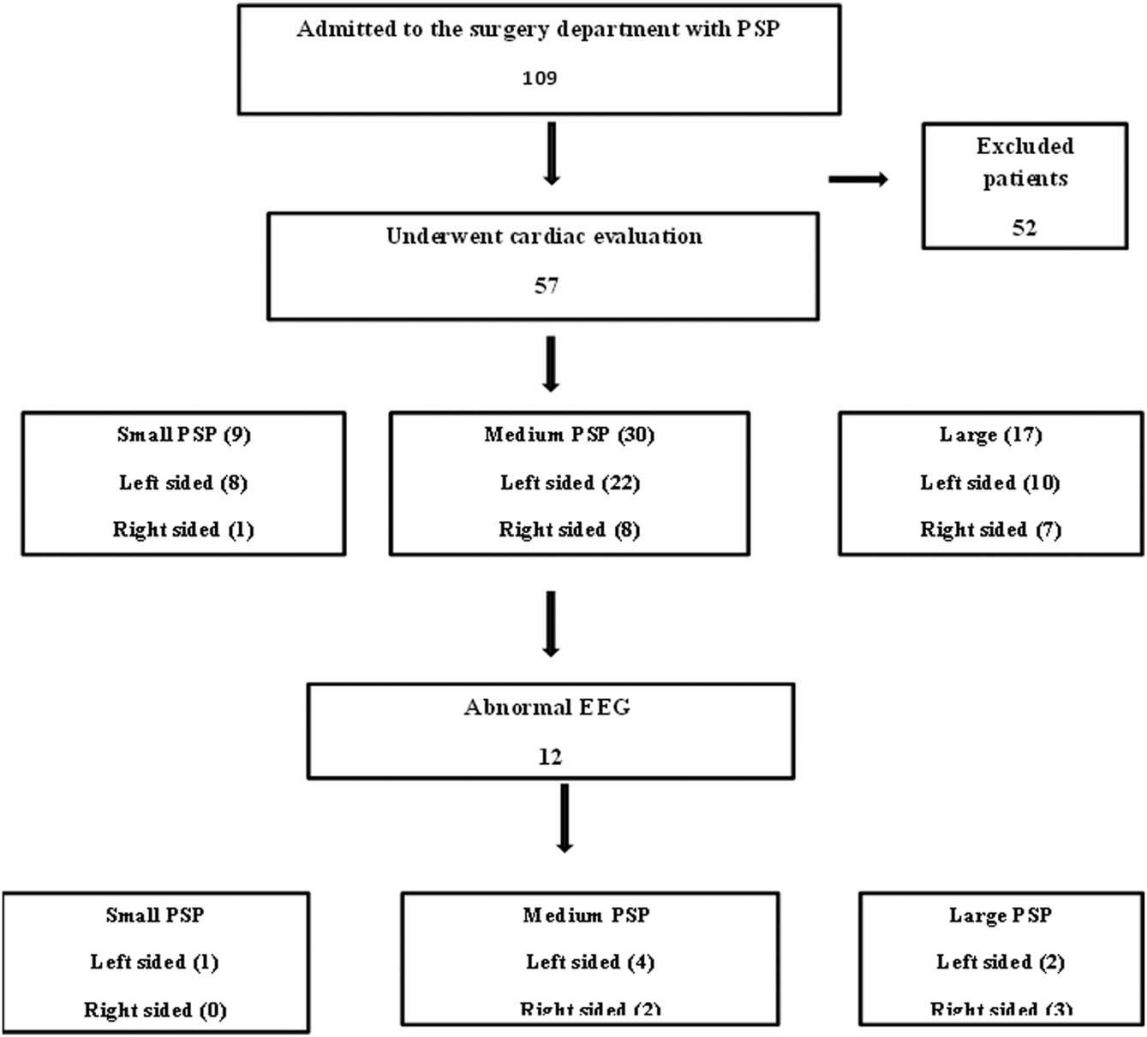
Flowchart of the study.

The pneumothorax size, according to the Collins equation, varied between 5% to 100%. Small right and left PSP were observed in 1 and 8 patients respectively. Medium and large PSP were observed in 30 and 17 respectively; of them, 8 and 7 were medium and large right sided, 22 and 10 left sided (Fig. 1). One patient was presented with medium bilateral pneumothorax. Serum Troponin-T levels were normal in all the participants (<0.01 ng/mL). ECG findings were normal in 45 (79%) patients and abnormal in 12 (21%) (Fig. 1). Abnormal findings observed included ST segment elevation in 5 patients, inverted T wave in 2 patients, incomplete right bundle branch block (RBBB) in 2 patients, poor R wave progression, left axis deviation and low QRS voltage in 1 patient each (Table 2).

**Table 2:** Correlation between size, side of pneumothorax and abnormal cardiac findings.

Patient	Age (years)	Gender	PNX (%)	PNX side	ECHO	ECG
1	17	Male	40	Right	normal	ST elevation V3–5
2	16	Female	30	Left	Normal	Poor R Wave Progression, Flattened T wave V1–6.
3	16	Male	84	Right	normal	ST elevation V2–6
4	16	Male	70	Right	normal	ST elevation V3–5
5	14	Male	59	Left	normal	Inverted T wave V1–6
6	17	Male	44	Left	normal	Left axis deviation -90
7	17	Male	28	Right	normal	ST elevation V2–6
8	16	Male	19	Left	normal	ST elevation II, III, AVF
9	16	Male	58	Right	normal	Incomplete RBBB
10	16	Male	27	Left	normal	WPW pattern, Inverted T wave II and III
11	17	Male	50	Left	normal	Low QRS Voltage
12	15	Male	100	Left	normal	Incomplete RBBB
13	16	Male	19		Minimal mitral and tricuspid regurgitation	Normal
14	17	Male	30	Left	Mitral valve prolapse	Normal

Abnormal echocardiographic findings were found in 2 patients (4.5%), from 44 performed examinations. One patient had a mitral valve prolapse and the other mild mitral and tricuspid regurgitation (Table 2).

Table 3 illustrates correlations between cardiac findings with other variables; no correlation was observed between groups (with or without cardiac findings) regarding age, gender, side and the dimension of pneumothoraxes, hospitalization length and follow-up period. No records of cardiovascular disturbances were mentioned during the hospitalization or follow up period.

**Table 3:** comparison between groups.

	Normal cardiac finding	Abnormal cardiac finding ^α^	*P* value	Total
Gender (female/male)	5/42 (11.9%)	2/14 (14.3%)	1.0	8/57 (14%)
Age, yr (mean)	15.6 (±0.8)	15.7 (±1.0)	.720	15.9 (±1.7)
Pneumothorax size:				
≤20%	8/41 (19.5%)	2/14 (14.3%)	.841	10/55 (18.2%)
21%–50%	22/41 (53.7%)	7/14 (50%)		29/55 (52.7%)
≥50%	11/41 (26.8%)	5/14 (35.7%)		16/55 (29.1%)
Pneumothorax size:				
≤20%	8/41 (19.5%)	2/14 (14.3%)	1.0	10/55 (18.2%)
>20%	33/41 (80.5%)	12/14 (85.7%)		45/55 (81.8%)
Pneumothorax side (left/right)	31/42 (73.8%)	8/14 (57.1%)	.480	39/56 (69.6%)
Hospitalization, days (median, IQR)	5 (IQR: 4–8)	5 (3.75–5.25)	.370	5 (4–7.25) n = 54
Follow up, mo (median)	2 (IQR: 1–12.25)	3.5 (IQR 1.-28.25)	.326	3 (IQR:1–14) n = 57

## 4 Discussion

ECG abnormalities among adults with PSP were reported in the past. We could not find similar works in the pediatric population and therefore decided to investigate the prevalence of ECG abnormalities in a group of pediatric patients with PSP.

The majority of participants were male and had left sided medium pneumothoraxes. Our results indicate that ECG changes in pediatric patients with PSP are not uncommon, presenting in 21% of them. The ECG findings included ST-segment elevation, inverted or flattened T wave, incomplete right bundle branch block, poor R wave progression, left axis deviation and low QRS voltage. No correlation was observed between the ECG findings and pneumothorax side or dimension. Echocardiography revealed normal findings in the majority of cases and serum Troponin-T levels were normal in all them.

Waltson et al^[4]^ studied 7 patients with left sided PSP and listed 4 ECG changes: rightward QRS deviation, precordial T wave inversion, decreased QRS amplitude and diminution of precordial R- voltage. Subsequently, electrical voltage alteration was described.^[4,15]^ Waltson et al proposed several mechanisms involved in ECG changes among PSP patients, rightward shift of QRS axis may be due to a heart rotation around its longitudinal axis and/or sudden increase in pulmonary vascular resistance causing right ventricular dilatation. Diminution of precordial R- voltage may be due to reduced conductance of the electrical impulse by retrosternal free air and posterior displacement of the heart. Similar ECG findings were observed in the current study regardless the pneumothorax side or dimension suggesting additional or other factors that might be involved in ECG changes during PSP.

Few reports exist of ST-segment elevation or ECG changes suggestive of acute myocardial infarction involving mostly older patients with medical history of coronary heart disease.^[6,16–20]^ However, a case of a young man with unremarkable medical background, presenting with left-sided PSP and ECG changes that included ST-segment elevations mimicking anterior myocardial infarction was reported.^[21]^ Some authors attributed ST-segment changes to transient coronary ischemia due to associated hypoxia and hypotension induced by mediastinal displacement. Krenke et al^[11]^ review the ECGs of 40 adult patients presented with PSP and found only 1 case with T-wave inversion. However, in the current study, 5 patients had ST-segment elevation; we believe none of them had evidence of myocardial injury as manifested by normal levels of serum Troponin-T and normal cardiac echocardiography. Troponin-T is a sensitive and specific marker in evaluating myocardial injury, but its role in healthy adolescents undergoing a reversible stress such as episode of PSP is unclear. One previous study examined Troponin serum levels in patients with stable coronary artery disease undergoing stress myocardial perfusion imaging and showed that Troponin concentrations were not closely associated with reversible myocardial ischemia.^[22]^ This may partially explain our results.

Interestingly, 4 of the 5 patients with ST elevation had right sided pneumothorax. Krenke et al reviewed 40 patients with spontaneous pneumothoraxes, both right and left-sided and found only 1 patient with T-wave inversion and no patients with ST-segment elevation.^[11]^ Another study that described ECG findings in PSP among 45 adult patients (20 right sided PSP); ST elevation was observed in 14 participants 8 of them had right sided PSP.^[23]^

Two participants in the present study had incomplete RBBB, one left sided pneumothorax and the second was right sided. Complete and incomplete RBBB were previously reported.^[11,24,25]^ RBBB is one of the findings on ECG with prevalence of 2.3% in the general population according to National Health Survey III in the United States.^[26]^ Cor-pulmonal can be one of the pathological conditions that can cause RBBB. It is possible that mechanisms involved in cases of PSP that can cause RBBB are related to raising the intrathoracic pressure and subsequently leading to increased pulmonary artery resistance which causes right ventricle strain resulting in defects of the conduction pathway.^[27,28]^

### 4.1 Limitations

This study has limitations due to its retrospective nature and the relative small number of participants. In addition no ECG records were found at discharge. Yet, as far as we know, this study is the first that describes serial cases of cardiac findings among children presenting with PSP. Our research group plans to carry out a prospective study in collaboration with other medical centers in order to better characterize the role of the ECG in pediatric patients represented with PSP and whether the side or size of PSP significantly presenting affect the pattern of ECG changes.

## 5 Conclusion

ECG changes were found in 21% among pediatric patients with PSP. No correlation was observed between ECG changes and side/size of pneumothorax. It is important to rule out pneumothorax among children presented with chest pain, dyspnea and ECG changes.

## Author contributions

**Conceptualization:** Baruch Klin, Haim Bibi, Ibrahim Abu Kishk.

**Data curation:** Baruch Klin, Haim Bibi.

**Formal analysis:** Itai Gueta.

**Investigation:** Ibrahim Abu Kishk.

**Methodology:** Baruch Klin, Itai Gueta.

**Supervision:** Itai Gueta, Haim Bibi, Ibrahim Abu Kishk.

**Validation:** Baruch Klin.

**Writing – original draft:** Baruch Klin, Ibrahim Abu Kishk.

**Writing – review & editing:** Baruch Klin.
